# L-shaped nonlinear relationship between magnesium intake from diet and supplements and the risk of diabetic nephropathy: a cross-sectional study

**DOI:** 10.3389/fnut.2025.1601338

**Published:** 2025-07-11

**Authors:** Jia Du, Xuetong Zhu, Ze Li, Qi Sun, Miao Sun, Heyuan Guan, Jiancheng Xu

**Affiliations:** Department of Laboratory Medicine, First Hospital of Jilin University, Changchun, China

**Keywords:** magnesium intake, diabetic nephropathy, dose–response relationship, threshold value, piecewise regression, Boruta algorithm

## Abstract

**Background and objectives:**

Given the ongoing controversy regarding the relationship between magnesium and diabetic nephropathy (DN), this study systematically evaluate the association between total magnesium intake from both dietary and supplemental sources and the risk of DN, and further explore its potential nonlinear dose–response pattern and threshold effect.

**Methods:**

Data were from the National Health and Nutrition Examination Survey 2007–2018. A multi-step analytical strategy was adopted: (1) confounders were selected using variance inflation factor and Boruta feature selection algorithm; (2) weighted multivariable logistic regression assessed the association between magnesium intake and DN; (3) restricted cubic splines (RCS), generalized additive models (GAM), and curve fitting were used to evaluate nonlinear dose–response trends; (4) piecewise regression identified potential thresholds; (5) subgroup analyses examined interactions across age, gender, BMI, hypertension, and cardiovascular disease.

**Results:**

A total of 3,355 participants were included (DN group: *n* = 1,295; non-DN group: *n* = 2,060). The magnesium intake among DN patients was significantly lower than that of non-DN patients [300 ± 171 mg/day vs. 329 ± 161 mg/day, *p* < 0.001]. After adjusting for confounders, each standard deviation (SD) increase in magnesium intake was associated with a 19% reduction in DN risk (OR = 0.81, 95% CI: 0.73–0.89). Compared with the lowest quartile of magnesium intake (Q1), the highest quartile (Q4) showed a significantly lower risk of DN (OR = 0.54, *p* < 0.001). RCS analysis suggested an L-shaped nonlinear association (nonlinearity-*p* = 0.003), which was further supported by GAM results. Piecewise regression analysis identified a turning point at 345.00 mg/day. Below this value, higher magnesium intake was significantly associated with lower DN risk; above this threshold, the protective effect plateaued. No significant interactions were found in the subgroup analyses.

**Conclusion:**

Total magnesium intake was inversely associated with DN risk, with a threshold identified at 345.00 mg/day. Below this level, increases in magnesium intake were significantly associated with reduced DN risk, whereas above this level, additional magnesium intake was not significantly associated with further reductions in DN risk. These findings provide new epidemiological evidence to inform magnesium intake recommendations and DN prevention strategies.

## 1 Introduction

Diabetic nephropathy (DN) is one of the most common microvascular complications in patients with diabetes. Epidemiological studies have shown that approximately 30 to 40% of diabetic individuals eventually develop DN (1, 2). With the global prevalence of diabetes continuing to rise, the incidence of DN-related end-stage renal disease (ESRD) has increased year by year, making it a leading cause of dialysis and placing a heavy burden on healthcare systems and the broader economy (3). Therefore, developing new and cost-effective strategies to delay or prevent the onset and progression of DN holds significant clinical and societal importance.

In recent years, magnesium has attracted considerable attention due to its diverse biological functions. As an essential mineral and a critical cofactor for more than 300 enzymes, magnesium plays an important role in various fundamental metabolic and physiological processes (4, 5). Studies have shown that magnesium is involved in maintaining metabolic homeostasis and regulating inflammation—processes that are closely linked to the onset and progression of chronic diabetic complications (6). Given its safety, affordability, and accessibility, the potential role of magnesium in the prevention and management of DN has garnered increasing interest. An epidemiological survey revealed that serum magnesium levels in diabetic patients were significantly lower than those in healthy individuals and tended to decline further during the progression of DN, suggesting that hypomagnesemia may be associated with the onset and worsening of DN (7). Another study reported that hypomagnesemia increased the risk of progression to ESRD in patients with DN, independent of other known risk factors for renal function deterioration (8). However, a study by Rezaei et al. found no significant association between serum magnesium concentrations and urinary albumin-to-creatinine ratio (UACR) (9). Given that UACR is an important clinical marker of early glomerular injury in DN, this finding suggests that serum magnesium may not be a reliable indicator for assessing DN risk. To date, the evidence regarding the relationship between serum magnesium and DN risk remains inconsistent.

It is worth noting that serum magnesium, a commonly used clinical measure, reflects only a small fraction of total body magnesium (approximately 0.3%), and may not accurately represent whole-body magnesium status (10). In contrast, assessing total magnesium intake offers a more comprehensive indication of individual magnesium exposure and may be more informative in evaluating the risk of chronic diseases. Based on this, we utilized large-scale, multi-cycle data from the National Health and Nutrition Examination Survey (NHANES) and assessed total magnesium intake from both dietary and supplemental sources as a composite indicator of exposure. We systematically evaluated its association with DN risk and further explored the dose–response relationship and potential threshold effects. These findings contribute to the epidemiological evidence base on magnesium intake and DN risk and may inform future mechanistic research and intervention strategies.

## 2 Methods

### 2.1 Data source and study population

This cross-sectional study was based on publicly available, de-identified data from NHANES (2007–2018). A total of 34,770 adults aged ≥ 20 years were included. NHANES data collection was approved by an institutional review board, and all participants provided written informed consent. The analytical dataset included demographic characteristics, dietary intake, physical examination results, laboratory measurements, and questionnaire responses.

Given that the primary objective of this study was to evaluate the association between magnesium intake and DN, only individuals with diagnosed diabetes were eligible for inclusion. Participants were excluded if they: (a) did not have diabetes; (b) lacked DN-related information; (c) had missing data on magnesium intake or key covariates; (d) reported implausible total energy intake (<500 or >5,000 kcal/day), as defined in previous studies (11).

**Figure 1:**
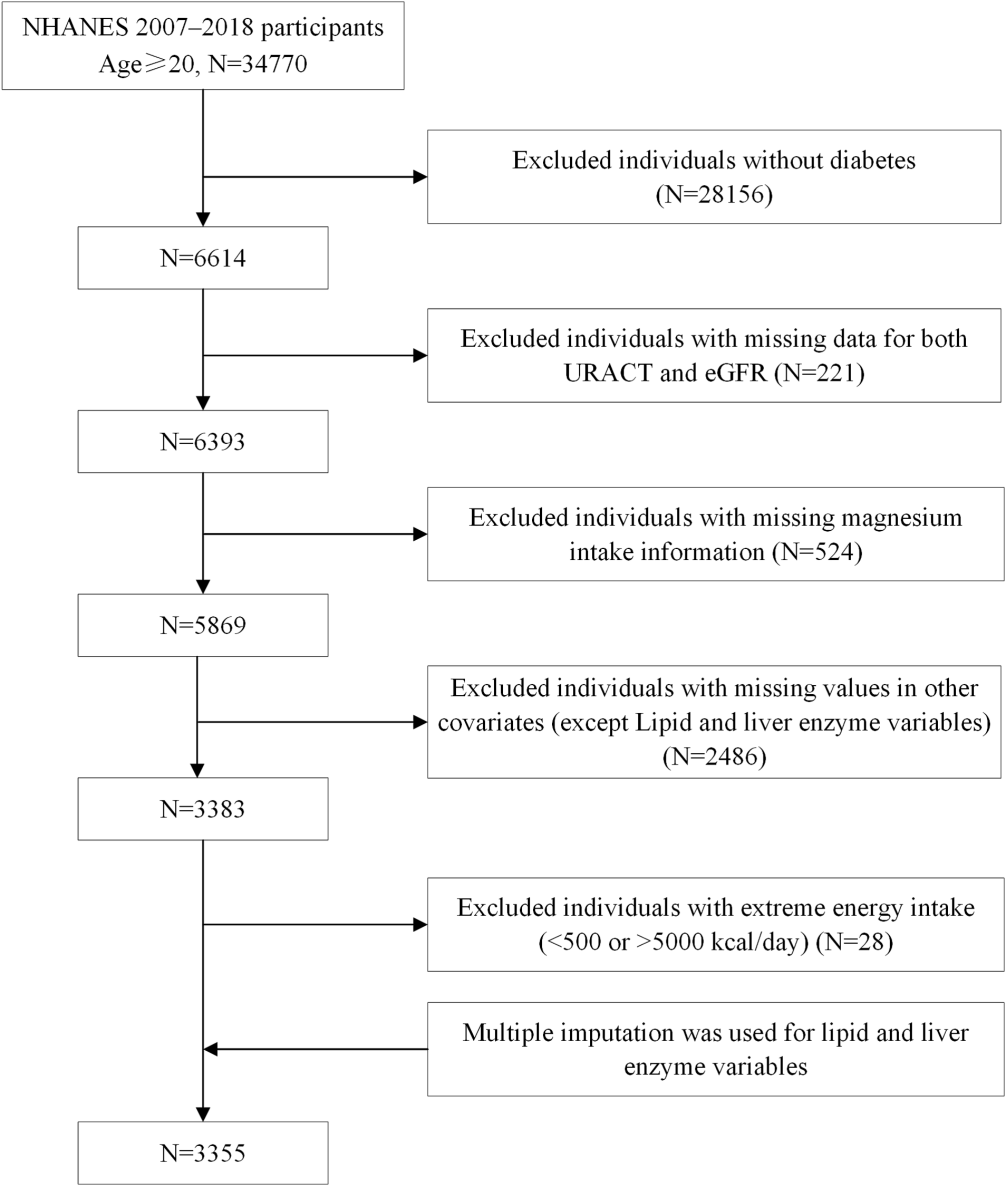
Flowchart of participant enrollment.

After initial screening, several metabolic indicators still had missing values, including triglycerides (TG), total cholesterol (TC), aspartate aminotransferase (AST), alanine aminotransferase (ALT), high-density lipoprotein cholesterol (HDL-C), and low-density lipoprotein cholesterol (LDL-C). To address these, we applied multiple imputation by chained equations (MICE) using stepwise regression. The imputation model incorporated all primary analytical variables and relevant covariates. Five complete datasets were generated after five iterations of chained equations, and one was selected for the primary analysis. After the aforementioned exclusion and imputation procedures, the final analytical dataset contained no missing data across any variables (Figure 1).

### 2.2 Assessment of diabetes and DN

According to the diagnostic criteria defined by the American Diabetes Association (ADA) (12), diabetes was identified based on any of the following: (i) fasting plasma glucose (FPG) ≥ 126 mg/dL (7.0 mmol/L); (ii) 2-h plasma glucose (2 h PG) ≥ 200 mg/dL (11.1 mmol/L) during an oral glucose tolerance test (OGTT); or (iii) glycated hemoglobin (HbA1c) ≥ 6.5% (48 mmol/mol). In addition, participants’ self-reported information was incorporated. Individuals who reported having been diagnosed with diabetes by a physician or who were currently taking glucose-lowering medications (including oral agents or insulin) were also classified as having diabetes.

DN was defined as either a urinary albumin-to-creatinine ratio (UACR) ≥ 30 mg/g or an estimated glomerular filtration rate (eGFR) < 60 mL/min/1.73 m^2^. In the NHANES dataset, the UACR for the 2007–2008 cycle was calculated from separate measurements of urinary albumin and urinary creatinine. From 2009 onward, UACR values were directly provided by the dataset. The eGFR was calculated based on serum creatinine (Scr). The CKD-EPI equation, updated in 2021 to remove the race coefficient for improved generalizability (13), was used for eGFR estimation. The formula is as follows:


$$\begin{array}{l} eGFR_{cr}=142\times \min{\left(\mathrm{Scr}/\kappa ,1\right)}^{\alpha }\times \max{\left(\mathrm{Scr}/\kappa ,1\right)}^{-1.200}\\ \;\;\;\;\;\;\;\;\;\;\;\;\;\;\;\;\;\;\;\times 0.9938^{\mathrm{Age}}\times 1.012^{{\left(\mathrm{if Female}\right)}_{\leftarrow }} \end{array}$$

*κ* represents a gender-specific standardized constant: 0.7 for females and 0.9 for males, *α* is a constant with values of −0.241 for females and −0.302 for males, and the term 1.012 ^(if female)^ represents the gender correction factor, equal to 1.012 for females and 1 for males.

### 2.3 Assessment of total magnesium intake

Total magnesium intake was defined as the sum of dietary magnesium (from food and beverages) and supplemental magnesium (from vitamins, minerals, or over-the-counter antacids). Both components were assessed using two 24-h dietary recalls: the first was conducted in person at a mobile examination center (MEC), and the second was completed by telephone 3 to 10 days later. Only participants who completed the dietary recall were subsequently asked about their use of dietary supplements. For each recall, magnesium intake from the previous day was recorded. The average intake from the two recalls was calculated separately for dietary and supplemental magnesium; if only one recall was available, that value was used. Accordingly, total magnesium intake was calculated as: (mean dietary magnesium from two recalls) + (mean supplemental magnesium from two recalls).

### 2.4 Covariate

Covariates included age, gender, body mass index (BMI), race, family income-to-poverty ratio (PIR), education level, and duration of diabetes (≤ 10 years or >10 years). Smoking status was categorized into three groups: never smokers (fewer than 100 cigarettes in their lifetime), former smokers (more than 100 cigarettes but not currently smoking), and current smokers (more than 100 cigarettes and currently smoking). Alcohol consumption was classified according to the Alcohol Use Questionnaire (ALQ) into current moderate drinkers, current heavy drinkers, former drinkers, and lifetime abstainers. Moderate and heavy drinking were defined according to daily alcohol intake. The definitions of former drinkers and lifetime abstainers varied slightly depending on survey years: for 2007–2016, classification was based on lifetime alcohol consumption and recent drinking frequency; for 2017–2018, classification relied on whether the participant had ever consumed alcohol and whether they had done so in the past year (see Supplementary Figure S1 for detailed definitions). Hypertension was defined as either (1) self-reported physician-diagnosed hypertension or (2) systolic blood pressure ≥140 mmHg or diastolic blood pressure ≥ 90 mmHg. Cardiovascular disease (CVD) was defined as self-reported history of any of the following: congestive heart failure, coronary heart disease, angina, myocardial infarction, or stroke (14). Cancer was defined as any type of cancer ever diagnosed by a clinician and self-reported by the participant. Depressive symptoms were assessed using the Patient Health Questionnaire-9 (PHQ-9), with scores ≥10 considered indicative of clinically significant depressive symptoms (15). Biochemical indicators included HDL-C, LDL-C, TG, TC, AST, and ALT. Calcium intake was calculated using the same approach as for magnesium. Energy intake was derived as the mean of two 24 h dietary recalls.

### 2.5 Statistical analysis

This study used WTDRD1, SDMVPSU, and SDMVSTRA from the NHANES dataset as the sample weights, primary sampling units, and stratification variables, respectively. Group differences in continuous variables were assessed using *t*-tests or Kruskal-Wallis tests, and results were expressed as means with standard deviations. Categorical variables were analyzed using chi-square tests, with unweighted frequencies and weighted percentages reported.

**Figure 2:**
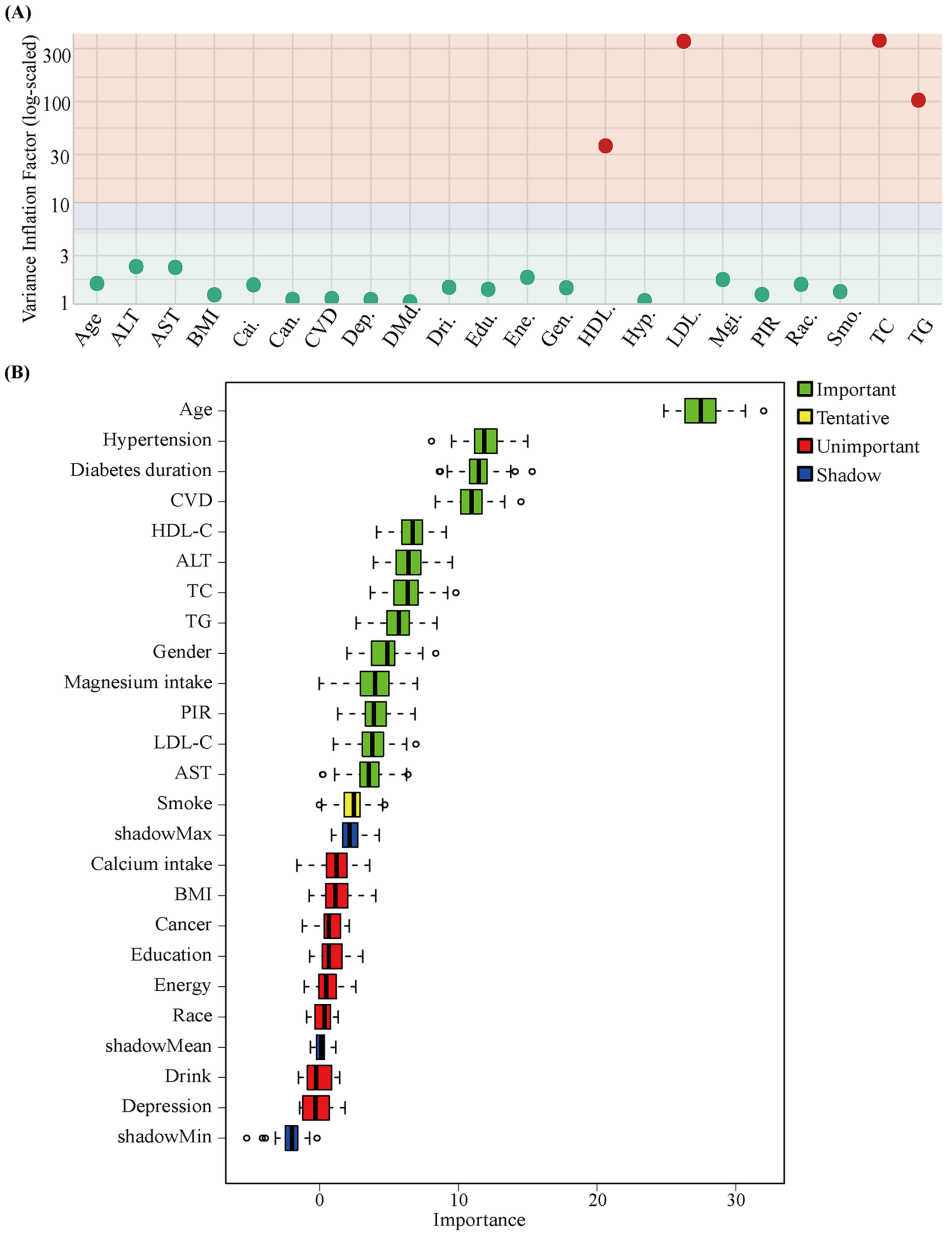
Results of variable collinearity assessment and Boruta feature selection. **(A)** Distribution of variance inflation factor (VIF) values across variables: green dots indicate low-collinearity variables (VIF < 5), red dots indicate variables with high collinearity (VIF > 10). The background shading reflects the degree of collinearity. Variables on the x-axis are labeled using short tags. Among them: Cai. = Calcium intake, Can. = Cancer, Dep. = Depression, DMd. = Diabetes duration, Dri. = Drinking status, Edu. = Education, Ene. = Energy intake, Gen. = Gender, HDL. = HDL-C, Hyp. = Hypertension, LDL. = LDL-C, Mgi. = Magnesium intake, Rac. = Race, Smo. = Smoke. **(B)** Boruta feature selection results: the box plot shows the distribution of variable importance, and different colors correspond to importance categories.

To evaluate the association between magnesium intake and DN, we employed weighted generalized linear logistic regression and multivariable logistic regression models. (1) Exposure variable characterization: Magnesium intake was represented in three forms: a standardized *z*-score continuous variable, a four-category variable, and an ordinal variable. The categorical variable was based on weighted quartiles of magnesium intake in the study population and divided into four groups: < 215 mg/day, 215–287 mg/day, 287–382 mg/day, and > 382 mg/day. The ordinal variable assigned rank scores to each group in the above order and was used for trend analysis. (2) Covariate selection and screening: Multicollinearity was assessed using the variance inflation factor (VIF > 5) and evaluated in combination with clinical relevance and statistical significance to determine inclusion in the model (see Figure 2). Among highly collinear lipid variables, only HDL-C was retained in the final multivariable model, while LDL-C, TG, and TC were excluded. The Boruta algorithm was then applied to identify statistically significant predictors by comparing variable importance against that of randomly generated “shadow” variables. Additionally, BMI was retained as a theoretically important covariate related to metabolic health. (3) Model construction strategy: Based on the above screening process, three logistic regression models were built: Model 1 included only magnesium intake; Model 2 further adjusted for gender, age, and BMI; and Model 3 additionally controlled for hypertension, diabetes duration, CVD, HDL-C, ALT, AST, PIR, and smoking status.

To evaluate the potential nonlinear relationship between magnesium intake and DN, restricted cubic splines (RCS), generalized additive models (GAM), and smooth curve fitting analyses were conducted, following the three-stage covariate adjustment strategy described above. The number of knots in the RCS models was determined based on the Akaike Information Criterion (AIC) to balance model fit and complexity (16). By comparing the AIC values across models with different numbers of knots, the optimal number of knots was identified as 4, 4, and 3 for Models 1, 2, and 3, respectively. Piecewise regression was implemented using the segmented package in R, which introduced threshold values to divide the continuous exposure variable into distinct intervals and separately fit associations on either side of the threshold, thereby identifying potential inflection points. Model fit was assessed by likelihood ratio tests (LRT), with *p* < 0.05 considered statistically significant.

Subgroup analyses were performed using weighted logistic regression models. Magnesium intake was standardized using *z*-scores to eliminate the influence of measurement unit differences on the comparison of regression coefficients. The selection of stratification variables was based on clinically relevant risk factors for DN and statistically significant predictors identified by the Boruta algorithm, including age, gender, BMI, CVD, hypertension, diabetes duration, HDL-C, and smoking status. In the HDL-C subgroup analysis, participants were classified into normal and abnormal HDL-C groups based on gender-specific cut-off values (< 39 mg/dL for men and < 50 mg/dL for women) (17). All analyses were conducted using R software (version 4.2.2), and a two-sided *p* < 0.05 was considered statistically significant.

## 3 Results

### 3.1 Baseline characteristics

A total of 3,355 participants were included in this study, comprising 1,295 individuals (38.6%) in the DN group and 2,060 individuals (61.4%) in the non-DN group. As shown in Table 1, there were observable differences in clinical characteristics, demographic distribution, and lifestyle factors between the two groups. Compared with the non-DN group, the DN group exhibited a longer duration of diabetes, a higher prevalence of hypertension and CVD, and a trend toward lower HDL-C levels. Participants in the DN group were also more likely to be male, older in age, have a lower PIR, and have lower energy and calcium intake. In addition, magnesium intake was significantly lower in the DN group than in the non-DN group (300 ± 171 mg vs. 329 ± 161 mg; *p* < 0.001).

**Table 1:** Summary characteristics of participants by DN.

Characteristic	Overall *N* = 3355^a^	Non-DN *N* = 2060^a^	DN *N* = 1295^a^	*p*-value^b^
Age (years)				<0.001
<50	589 (19.9%)	439 (22.7%)	150 (14.7%)	
≥50	2,766 (80.1%)	1,621 (77.3%)	1,145 (85.3%)	
Gender				0.013
Male	1773 (52%)	1,013 (49%)	760 (58%)	
Female	1,582 (48%)	1,047 (51%)	535 (42%)	
BMI (kg/m^2^)				0.4
0-25	418 (11%)	242 (11%)	176 (11%)	
25–29.99	938 (25%)	602 (26%)	336 (23%)	
≥30	1999 (64%)	1,216 (63%)	783 (66%)	
Race				0.043
Non-Hispanic White	1,235 (63%)	742 (64%)	493 (61%)	
Non-Hispanic Black	879 (14%)	507 (13%)	372 (17%)	
Mexican American	567 (8.9%)	364 (8.7%)	203 (9.3%)	
Other Hispanic	349 (5.4%)	225 (5.6%)	124 (5.1%)	
Other	325 (8.5%)	222 (8.9%)	103 (7.7%)	
PIR				<0.001
<2.5	2,102 (49.79%)	1,217 (45.67%)	885 (57.70%)	
≥2.5	1,253 (50.21%)	843.00 (54.33%)	410 (42.30%)	
Education				0.087
Under high school	1,079 (22%)	631 (21%)	448 (25%)	
High school or equivalent	781 (26%)	477 (25%)	304 (27%)	
Above high school	1,495 (52%)	952 (54%)	543 (48%)	
Drink				0.15
Current heavy drinkers	643 (21%)	427 (22%)	216 (18%)	
Current moderate drinkers	893 (32%)	563 (32%)	330 (31%)	
Former drinkers	1,274 (33%)	734 (32%)	540 (36%)	
Lifetime abstainers	545 (14%)	336 (14%)	209 (15%)	
Smoke				0.021
Current smoke	528 (16%)	332 (17%)	196 (15%)	
Former smoke	1,186 (36%)	679 (33%)	507 (40%)	
Non smoke	1,641 (48%)	1,049 (50%)	592 (45%)	
Hypertension				<0.001
No	831 (25%)	623 (30%)	208 (17%)	
Yes	2,524 (75%)	1,437 (70%)	1,087 (83%)	
CVD				<0.001
No	2,413 (73%)	1,609 (78%)	804 (63%)	
Yes	942 (27%)	451 (22%)	491 (37%)	
Cancer				0.072
No	2,835 (82%)	1765 (84%)	1,070 (80%)	
Yes	520 (18%)	295 (16%)	225 (20%)	
Diabetes duration (years)				<0.001
≤10	1879 (59%)	1,285 (63%)	594 (50%)	
>10	1,476 (41%)	775 (37%)	701 (50%)	
Depression				0.4
Not significant	2,901 (87%)	1792 (87%)	1,109 (86%)	
Clinically significant	454 (13%)	268 (13%)	186 (14%)	
AST (U/L)	27 (22)	26 (18)	28 (30)	>0.9
ALT (U/L)	27 (38)	26 (16)	29 (61)	0.11
TG (mg/dl)	147 (141)	145 (136)	150 (151)	0.5
TC (mg/dl)	181 (46)	181 (45)	181 (49)	0.5
HDL-C (mg/dl)	47 (14)	47 (14)	45 (14)	0.005
LDL-C (mg/dl)	105 (46)	105 (44)	105 (48)	0.7
Energy (kcal/day)	1875 (721)	1906 (722)	1815 (714)	0.022
Calcium intake (mg/day)	1,070 (562)	1,098 (570)	1,016 (544)	<0.001
Magnesium intake (mg/day)	319 (165)	329 (161)	300 (171)	<0.001

a Median (IQR) for continuous; *n* (%) for categorical.

b Pearson’s X^2: Rao & Scott adjustment; Design-based Kruskal Wallis test.

BMI: body mass index; PIR: poverty income ratio; CVD: cardiovascular disease; AST: asparate aminotransferase; ALT: alanine aminotransferase; TG: triglycerides; TC: total cholesterol; HDL-C: high-density-lipoprotein cholesterol; LDL-C: low-density-lipoprotein cholesterol.

### 3.2 Associations between the magnesium intake and DN

Among all participants, an inverse association was observed between magnesium intake and the risk of DN (Table 2). Using the lowest quartile (Q1) as the reference, higher magnesium intake levels (Q3 and Q4) were significantly associated with a reduced risk of DN. In the unadjusted model (Model 1), the odds ratio (OR) for Q4 was 0.58 (95% CI: 0.44–0.76; *p* < 0.001). This association remained statistically significant after adjusting for age, gender, and body mass index (Model 2: OR = 0.49; 95% CI: 0.37–0.65; *p* < 0.001), and persisted in the fully adjusted model (Model 3: OR = 0.54; 95% CI: 0.42–0.70; *p* < 0.001). When treated as a continuous variable, each standard deviation increase in magnesium intake was associated with a 19% lower risk of DN (Model 3: OR = 0.81; 95% CI: 0.73–0.89; *p* < 0.001). Moreover, analysis based on the ordinal categorization of intake further supported a significant gradient association between increasing magnesium intake and decreasing DN risk (*P* for trend < 0.001).

**Table 2:** ORs (95% CIs) for DN based on magnesium intake.

Characteristic	Model 1			Model 2			Model 3		
	OR^a^	95%CI^a^	*P*-value	OR^a^	95%CI^a^	*P*-value	OR^a^	95%CI^a^	*P*-value
Continuous	0.83	0.75, 0.91	<0.001	0.78	0.70, 0.88	<0.001	0.81	0.73, 0.89	<0.001
Quartiles									
Q1	—	—		—	—		—	—	
Q2	0.86	0.69, 1.08	0.193	0.77	0.61, 0.97	0.031	0.84	0.66, 1.06	0.134
Q3	0.56	0.41, 0.76	<0.001	0.50	0.35, 0.70	<0.001	0.54	0.38, 0.77	<0.001
Q4	0.58	0.44, 0.76	<0.001	0.49	0.37, 0.65	<0.001	0.54	0.42, 0.70	<0.001
P for trend			<0.001			<0.001			<0.001

a OR: Odds Ratio, CI: Confidence Interval.

Model 1: Unadjusted. Model 2: Adjusted for Age, Gender, and BMI. Model 3: Adjusted for Age, Gender, BMI, Hypertension, Diabetes duration, CVD, HDL-C, ALT, AST, PIR and Smoke.

### 3.3 Dose–response and threshold effect of magnesium intake on DN

**Figure 3:**
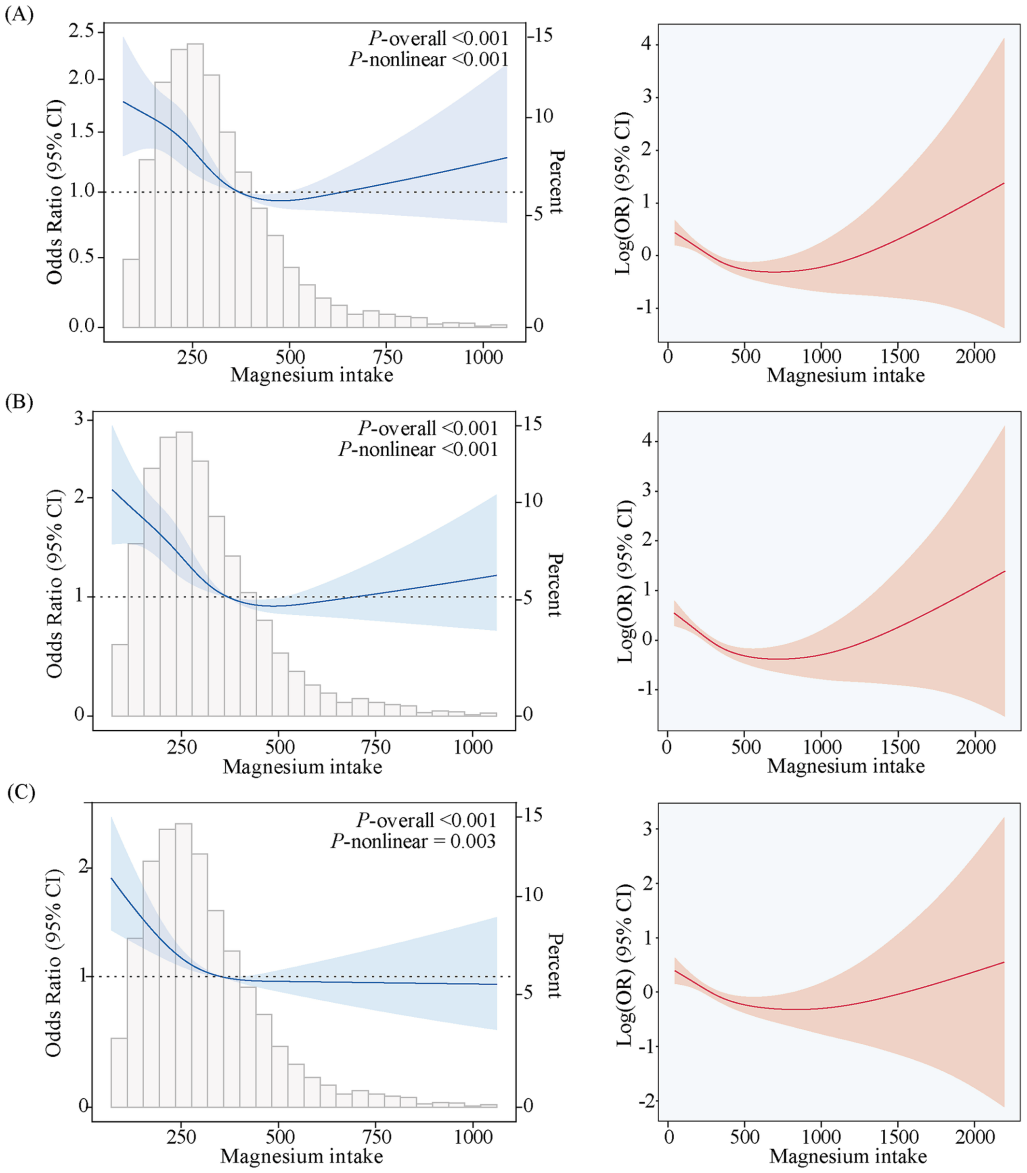
Restrictive cubic spline (RCS) and generalized additive model (GAM) analyses of magnesium intake and DN risk. Left: RCS analysis with blue curve representing odds ratios (OR) and shaded area indicating 95% CI, with histogram of participant distribution. Right: GAM analysis with red curve representing smooth fit and shaded area indicating 95% CI. **(A)** Unadjusted; **(B)** adjusted for age, gender, and BMI; **(C)** adjusted for age, gender, BMI, hypertension, diabetes duration, CVD, HDL-C, ALT, AST, PIR and smoke.

In the entire cohort, a significant nonlinear relationship was observed between magnesium intake and DN risk (*P*-nonlinear < 0.05; Figure 3). The unadjusted RCS curve suggested a U-shaped trend, whereas the fully adjusted RCS model (Model 3) showed a near L-shaped pattern. This nonlinear trend was further validated by smooth curve fitting and GAM. As covariates were progressively adjusted, the GAM curves stabilized, confirming the robustness of the nonlinear association.

Piecewise regression analysis revealed a clear threshold effect between magnesium intake and DN risk (Table 3). After adjusting for multiple covariates, the optimal inflection point was identified at 345.00 mg/day. Below this threshold, each 1 mg/day increase in magnesium intake was associated with a 0.2% reduction in DN risk (OR = 0.998, 95% CI: 0.996–0.999, *p* < 0.001). Above 345.00 mg/day, further magnesium intake was no longer significantly associated with a reduction in DN risk (*p* = 0.826). The likelihood ratio test (LRT) indicated that the piecewise model provided a significantly better fit than the restricted model (*p* = 0.004), supporting the presence of a threshold effect.

**Table 3:** Threshold effect analysis of magnesium intake on DN.

	Adjusted OR (95% CI)*	*P*-value
Fitting by standard logistic regression model	0.999 (0.999, 1.000)	<0.001
Fitting by piecewise logistic regression model		
Magnesium intake < 345.00 (mg/day)	0.998 (0.996, 0.999)	<0.001
Magnesium intake ≥ 345.00 (mg/day)	1.000 (0.999, 1.001)	0.826
Log likelihood ratio		0.004

*Adjusted for: gender, age, PIR, BMI, HDL-C, ALT, AST, smoke, hypertension, diabetes duration, CVD.

### 3.4 Subgroup analysis

**Figure 4:**
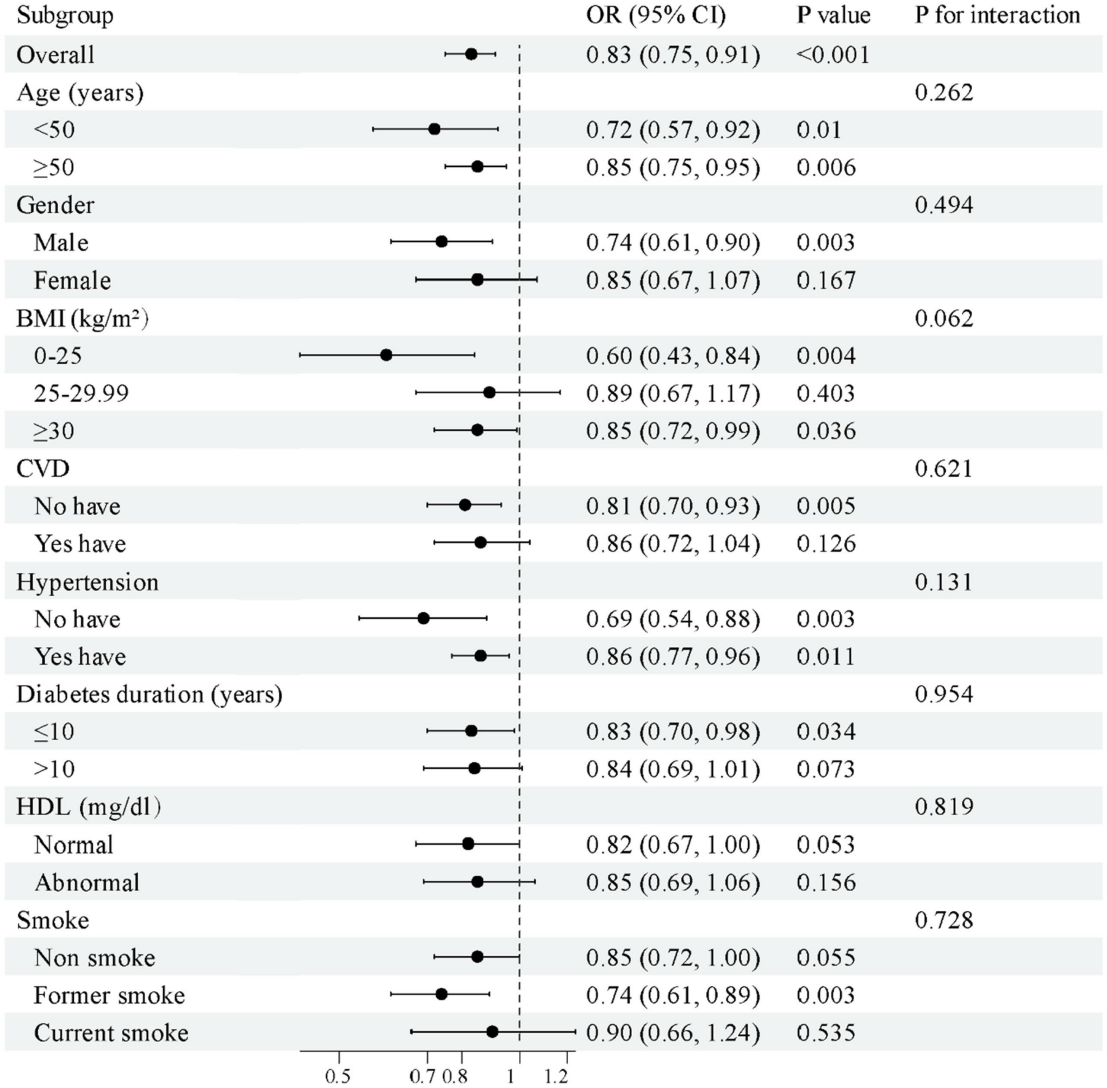
Subgroup analysis of magnesium intake and the risk of DN.

Subgroup analysis revealed a significant inverse association between magnesium intake and DN risk across various subgroups (Figure 4). The negative association was more pronounced in participants of all age groups, with or without hypertension, in men, those with BMI 0–25 or ≥ 30, those without CVD, with diabetes duration ≤ 10 years, with normal HDL-C levels, and former smokers. No significant interactions were observed across subgroups (*P* for interaction > 0.05 for all), indicating that these factors did not modify the relationship between magnesium intake and DN risk, thereby supporting the stability of the association across subgroups.

## 4 Discussion

In this cross-sectional analysis based on NHANES 2007–2018 data, a significant inverse association was observed between total magnesium intake and the risk of DN. Subgroup analyses showed that although the magnitude of this association varied across population subgroups, the overall protective trend remained consistent, with no significant interaction effects detected. Furthermore, analyses using RCS, smooth curve fitting, and GAM revealed an L-shaped nonlinear relationship between magnesium intake and DN risk. Piecewise regression analysis identified a threshold at approximately 345.00 mg/day. Below this threshold, each 1 mg/day increase in magnesium intake was associated with a 0.2% reduction in DN risk, whereas beyond this level, further increases in magnesium intake were not significantly associated with a further reduction in DN risk.

To accurately assess the relationship between total magnesium intake and DN risk, we constructed three logistic regression models with stepwise covariate adjustment. Variable selection was guided by VIF analysis and the Boruta algorithm, taking into account both statistical relevance and clinical plausibility. As shown in Figure 2, HDL-C, LDL-C, TG, and TC were highly collinear, reflecting both their physiological interdependence and the co-variation characteristic of dyslipidemia in individuals with metabolic syndrome (18). Although the lipid parameters are highly correlated, we retained one lipid variable in the model to represent lipid-metabolism status because of its clinical value. Boruta analysis indicated that all four lipid parameters were of significant importance for DN outcomes (Figure 2), with HDL-C receiving the highest importance score (detailed scores are provided in Supplementary Table S1). Therefore, HDL-C was incorporated as the representative lipid variable into the subsequent multivariable-adjusted models. VIF values for all variables included in the final model were below 5 (see Supplementary Figure S2), indicating acceptable levels of multicollinearity.

In this study, total magnesium intake was assessed by integrating dietary sources and supplemental intake. While magnesium is primarily obtained from foods such as nuts, seeds, whole grains, and leafy greens, changes in dietary patterns have resulted in suboptimal intake for many individuals (19). As a result, some people have turned to supplemental magnesium to meet recommended levels. According to the NHANES dietary survey protocol, participants who completed the dietary recall were further asked about their use of supplements. Among the 3,355 participants included in this study, 901 reported taking magnesium supplements in addition to dietary sources. These findings highlight the importance of jointly evaluating dietary and supplemental intake to accurately reflect individual magnesium exposure.

Magnesium participates in numerous metabolic and physiological functions within the human body. Research has indicated that it plays a key role in regulating insulin sensitivity and serves as a necessary cofactor for several enzymes involved in insulin signalling pathways (20). Magnesium deficiency may impair the activity of insulin receptor tyrosine kinase and downstream signalling, thereby promoting insulin resistance (21, 22). Given that insulin resistance and hyperinsulinemia are recognized as major contributors to DN development, improved insulin function may partially explain the observed inverse association between magnesium intake and DN risk (23). In addition to its effects on insulin metabolism, magnesium may also exert protective effects through antioxidant mechanisms (24). Although current research on the mechanisms of magnesium in DN is limited, existing evidence indicates that oxidative stress plays an important role in the pathogenesis of DN (25, 26). As an essential cofactor for various antioxidant enzymes, magnesium helps eliminate reactive oxygen species (ROS), promotes antioxidant enzyme activity, and enhances the expression of antioxidant proteins (27). In addition, magnesium is involved in the regulation of the renin–angiotensin–aldosterone system (RAAS). Studies have shown that insufficient magnesium intake can exacerbate Ang-II-induced aldosterone secretion and vasoconstriction, leading to glomerular hyperperfusion and hyperfiltration, whereas adequate magnesium intake helps suppress excessive RAAS activation and reduce renal hemodynamic stress (28). Therefore, increasing magnesium intake may benefit renal function and lower the risk of DN through multiple mechanisms, including improving insulin resistance, exerting antioxidant effects, and regulating hemodynamics.

Notably, we found an L-shaped dose–response relationship between total magnesium intake and the risk of DN, with a threshold at approximately 345.00 mg/day (see Table 3). Above this threshold, higher magnesium intake does not appear to provide additional renal protection, which may be attributable to the body’s physiological mechanisms for regulating magnesium absorption and excretion. Previous studies have shown that intestinal magnesium absorption is saturable: when intake is low, absorption rates can reach up to 80%, but as intake rises, these rates drop to about 20% (29). This effect is closely linked to the function of TRPM6/TRPM7 channels in intestinal epithelial cells, which mediate active magnesium transport. As intracellular magnesium levels increase, TRPM6 channel activity decreases, thereby limiting further absorption and preventing excess accumulation in the body (30, 31). In addition, there is a physiological limit to renal magnesium reabsorption. Renal tubules typically reabsorb about 95% of filtered magnesium to help maintain stable serum magnesium concentrations (32). When serum magnesium levels rise, the kidneys increase magnesium excretion by reducing reabsorption in the proximal and distal tubules, thereby preventing magnesium accumulation in the body (33). It is worth noting that an epidemiological study found that daily supplementation with 2.25 g of magnesium citrate (containing approximately 360 mg of elemental magnesium) could improve renal function indicators in patients with DN; however, more than half of the participants experienced mild gastrointestinal discomfort (34). Taken together, when magnesium intake exceeds 345.00 mg/day, the body likely maintains physiological magnesium homeostasis by adjusting both intestinal absorption and renal excretion, which may help explain why the renal protective effect of magnesium does not appear to increase further with higher intake. This physiological regulation offers a biological explanation for the observed plateau in DN risk reduction, suggesting that appropriate dosing should be considered in future magnesium supplementation strategies.

Subgroup analyses revealed that the protective effect of magnesium intake on DN was more pronounced among participants who were younger (OR = 0.72 vs. OR = 0.85, *p* < 0.05), had a shorter duration of diabetes, lower BMI, no CVD, normal HDL-C levels, or a history of former smoking. These groups generally have only mild renal impairment and relatively favorable metabolic profiles, which may improve the bioavailability of magnesium and thereby enhance its protective effect against DN. Notably, a previous study found that individuals with BMI ≥ 30 may have a greater need for magnesium due to underlying metabolic dysregulation and insulin resistance, and thus may be more likely to benefit from magnesium supplementation (35). However, in practice, obesity is often accompanied by chronic inflammation and other complex metabolic disorders, which may partially offset the potential benefits of magnesium. This may explain why the OR observed in the obese group (BMI ≥ 30, OR = 0.85) was higher than that in the normal-weight group (BMI 0–25, OR = 0.60), although the overall inverse trend remained consistent. Additionally, evidence suggests that men tend to have higher levels of inflammation and oxidative stress, possibly making them more responsive to the anti-inflammatory and antioxidant effects of magnesium (36). Overall, although the strength of the inverse association varied across subgroups, the general trend remained consistent (Figure 4). This supports a broad physiological basis for the protective role of magnesium intake in reducing DN risk. The magnitude of the effect may be influenced by metabolic health, inflammatory status, and comorbidity profile.

This study has several strengths. First, it utilized the nationally representative NHANES database, which employed a complex multistage probability sampling method covering individuals of different ages, sexes, and socioeconomic backgrounds. As a result, the statistical associations between magnesium intake and DN are more representative. Secondly, this study systematically revealed an L-shaped nonlinear dose–response relationship between total magnesium intake and the risk of DN, and proposed an optimal intake threshold. In addition, multiple rigorous statistical methods and machine learning–based variable selection were applied to effectively control bias and ensure the scientific validity of the models. Total magnesium intake was assessed by combining both dietary and supplemental sources, which better reflects the actual intake patterns of contemporary populations. Overall, the results of this study offer valuable evidence to support the prevention and nutritional management of DN and enrich the research in this field.

This study also has some limitations. First, for missing values in lipid and liver enzyme covariates, we applied multiple imputation to minimise the impact of missing covariate data on sample size and statistical power; it should be noted that the main analytic variables had no missing data and imputation was performed only for covariates. Although multiple imputation may introduce some degree of bias, sensitivity analyses restricted to participants with complete data for all variables yielded results consistent with the main analysis (Supplementary Table S2), suggesting that the main findings are robust. Second, the observational nature of the NHANES cross-sectional data allows us to identify associations but does not entirely exclude the possibility of reverse causality. Additionally, the assessment of dietary and supplemental magnesium intake relied on participants’ self-reported recall, which may have introduced recall bias and measurement error. Despite these limitations, our findings provide important insights for magnesium management and individualized interventions in patients with DN. Further prospective studies or randomized controlled trials are warranted to validate these results and inform clinical practice.

## 5 Conclusion

The results of this study clarified a significant inverse association between total magnesium intake and the risk of DN, with an L-shaped nonlinear dose–response relationship and a threshold of 345.00 mg/day. When magnesium intake was below 345.00 mg/day, increasing intake was associated with a substantial reduction in DN risk; above this threshold, the effect of additional magnesium intake on reducing DN risk was no longer statistically significant. These findings provide preliminary evidence to inform magnesium management and individualized intervention strategies for patients with DN.

## Data Availability

Publicly available datasets were analyzed in this study. This data can be found here: https://www.cdc.gov/nchs/nhanes/index.htm.
