# Supplementary material for: L-shaped nonlinear relationship between magnesium intake from diet and supplements and the risk of diabetic nephropathy: a cross-sectional study
Source: Front Nutr. 2025 Jul 11;12:1601338. doi: 10.3389/fnut.2025.1601338 (PMC12289502; doi:10.3389/fnut.2025.1601338)
Supplement: Supplementary file 1 [file Data_Sheet_1.docx]

**Fig. S1** Classification of drinking status into four groups based on the Alcohol Use Questionnaire and differences in data structure across survey years.


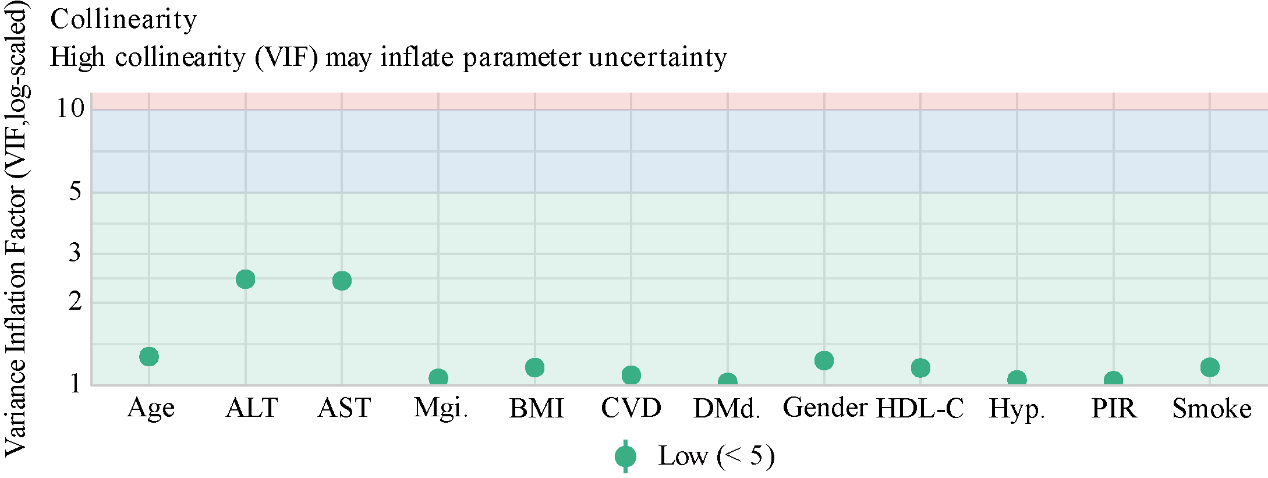


**Fig. S2** The distribution of variance inflation factors (VIFs) of all variables that were finally included in the adjustment calculation by the model. Green dots represent variables with low collinearity, red dots represent variables with VIF > 10, and the background color indicates the degree of collinearity, with the red area representing a high degree of collinearity. Variables on the x-axis are labeled using short tags. Among them: Mgi. = Magnesium intake, DMd. = Diabetes duration, Hyp. = Hypertension, PIR = Poverty income ratio, Smoke = Smoking status.

**Table S1** Variable selection results from the Boruta algorithm (maxRuns = 100).

| Variables | Mean Importance | Max Importance | Norm Hits | Decision |
| --- | --- | --- | --- | --- |
| Age | 27.50 | 32.01 | 1.00 | Confirmed |
| Hypertension | 11.97 | 14.99 | 1.00 | Confirmed |
| Diabetes duration | 11.51 | 15.31 | 1.00 | Confirmed |
| CVD | 10.94 | 14.51 | 1.00 | Confirmed |
| HDL-C | 6.73 | 9.11 | 1.00 | Confirmed |
| ALT | 6.53 | 9.55 | 1.00 | Confirmed |
| TC | 6.26 | 9.82 | 1.00 | Confirmed |
| TG | 5.67 | 8.45 | 1.00 | Confirmed |
| Gender | 4.68 | 8.36 | 0.95 | Confirmed |
| Magnesium intake | 4.03 | 7.03 | 0.84 | Confirmed |
| PIR | 3.98 | 6.87 | 0.86 | Confirmed |
| LDL-C | 3.75 | 6.93 | 0.87 | Confirmed |
| AST | 3.52 | 6.36 | 0.85 | Confirmed |
| Smoke | 2.35 | 4.69 | 0.53 | Tentative |
| Calcium intake | 1.15 | 3.59 | 0.14 | Rejected |
| BMI | 1.27 | 4.04 | 0.09 | Rejected |
| Cancer | 0.77 | 2.12 | 0.02 | Rejected |
| Education | 0.81 | 3.09 | 0.04 | Rejected |
| Energy | 0.55 | 2.60 | 0.03 | Rejected |
| Race | 0.26 | 1.32 | 0.00 | Rejected |
| Drink | -0.04 | 1.42 | 0.00 | Rejected |
| Depression | -0.19 | 1.83 | 0.00 | Rejected |

This table presents the results of variable selection using the Boruta algorithm, including the importance ranking and selection status of each variable. “Norm Hits” indicates the proportion of iterations in which a variable was identified as important, and “Decision” represents the final selection status (confirmed, tentative, or rejected).

**Table S2** Sensitivity analysis of the association between magnesium intake and DN risk based on data without multiple imputation

| Characteristic | **OR^1^** | **95%CI^1^** | ***P*-value** |
| --- | --- | --- | --- |
| Magnesium intake | 0.68 | 0.57, 0.81 | <0.001 |

^1^OR: Odds Ratio, CI: Confidence Interval.

Model adjusted for Age, Gender, BMI, Hypertension, Diabetes duration, CVD, HDL-C, ALT, AST, PIR, and smoking status.
